# Shape Dynamics of Bouncing Droplets

**DOI:** 10.1038/s41598-019-42580-5

**Published:** 2019-04-15

**Authors:** David V. Svintradze

**Affiliations:** School of Health Sciences, University of Georgia, Tbilisi, 0171 Georgia

## Abstract

Oscillating shape motion of a freely falling and bouncing water droplet has long fascinated and inspired scientists. We propose dynamic non-linear equations for closed, two-dimensional surfaces in gravity and apply it to analyze shape dynamics of freely falling and bouncing drops. The analytic and numerical solutions qualitatively well explain why drops oscillate among prolate/oblate morphology and display a number of features consistent with experiments. In addition, numerical solutions for simplified equations indicate nonlinear effects of nonperiodic/asymmetric motion and the growing amplitude in the surface density oscillations and well agree to previous experimental data.

## Introduction

Droplets are very peculiar systems mostly due to their shape dynamic properties^1^. They conserve the shape at the rest due to a surface tension, but are very deformable. Small perturbation of an equilibrium shape may induce large deformations in surface morphologies. One can trigger droplet inertial motion as well as morphological dynamics by inducing substrate surface oscillation^2^. Gradient of free substrate surface energy or angle difference between droplets leading and trailing edges provokes self-propulsion^3,4^. Droplets dynamical properties and their vibration modes may control their motion over substrate surface^5,6^ and transiently defy gravity^7^. The contact with the substrate can be minimized by the development of super hydrophobic surfaces^8^, allowing droplets to bounce on the surface like elastic balls^9^.

Recent experiments demonstrate that an oscillating surface can launch wobbly water drop into the air at higher speed than it would launch a hard ball of the same mass^2^. Therefore, a synchronization between the internal vibration of a drop projectile and the frequency of the rising and falling surface can more than double kinetic energy of the droplets^2^.

The behavior of bubbles differs from drops in gas-liquid systems. They display much broad range of shape deformations including turbulence^10^, thickness variations^11–13^, the Marangoni effect^14^, draining^15^, and ejection of droplets^16^, rupture^17^, self-adaptation^18^, and chaotic behavior^19^. From this broad range of effects, numerical solutions of dynamic nonlinear equations for free thin fluid films explained thickness variations^20^.

Despite of numerous experimental data^1–9^, description of droplets shape dynamics remain analytically largely unsolved. Some dynamic effects in linear regimes have led to the classical wave equations^21,22^. Numerical simulations of Navier-Stokes proved to be effective tool in the path of describing shape motions of drops and bubbles^23–25^. Furthermore, numerical solution of one-dimensional Navier-Stokes equations showed that simulated shape dynamics agrees with experiment qualitatively^26^. In addition, combination of experiments and numerical simulations explained the shapes of singularities around the neck for a drop falling from a faucet^27^.

In the letter, we apply closed, two-dimensional surface dynamics equations^28,29^ and analytically address dynamic morphological patterns of droplets observed in experiments^1–9^. Our theoretical model explains why droplets can transiently defy gravity. Even though the results well agree with previous ones, the formalism we apply is new and different from previous theories. The purpose of the paper is to show the effectiveness of the differential variational surface (DVS) formalism in handling the shape dynamic problems and pinpoint existence of relatively simple dynamic solutions. We discuss shape dynamics of droplets, though due to generality of our dynamic solutions the analyses can be extended to wobbly dynamics of bubbles too.

## Theory

To address shape dynamics properties of droplets, instead of using classically well-defined fluid dynamics (or we shall call it Navier-Stokes dynamics) we examine the surface dynamics of droplets. As far as the surface is closed and two-dimensional it has to follow to our generic equations of surface motions^29^. In this section, we briefly review fundamental principles of the equations. We base on the extension of differential geometry for moving surfaces. Because of the surface is moving, all parameters: base vectors ***S***_***i***_ (Vectors are designated by bold letters throughout the paper), the surface area *S*, the surface topology, the enclosed volume Ω are all time dependent functions.

Procedures for defining base vectors, metric tensor, and contravariant/covariant derivatives follow fundamentals of differential geometry. The base vectors are defined as partial derivative of the position vector ***S***_***i***_ = ∂_*i*_***R*** = ∂***R***/∂*S*^*i*^, *i* = 1, 2, where *S*^*i*^, ***R*** are the surface coordinate for an arbitrary chosen point and the position vector.

The *α* = 1, 2, 3 component of the surface velocity ***V*** is defined as *V*^*α*^ = ∂*X*^*α*^/∂*t* (*X*^*α*^ is a space coordinate for the point on the surface). The surface velocity has the normal *C* and tangential *V*^*i*^ velocities, so that

1 $${\boldsymbol{V}}=C{\boldsymbol{N}}+{V}^{i}{{\boldsymbol{S}}}_{{\boldsymbol{i}}}$$

where *V*^*i*^***S***_***i***_ = *V*^1^***S***_**1**_ + *V*^2^***S***_**2**_ implies Einstein summation convention throughout the text: repeated indexes in upper and down indexes is shortly written summation by the index.

Definitions of the surface velocity and the base vectors form fundamentals for defining Christoffel symbols ${{\rm{\Gamma }}}_{jk}^{i}={{\boldsymbol{S}}}^{{\boldsymbol{i}}}\cdot {\partial }_{j}{{\boldsymbol{S}}}_{{\boldsymbol{k}}}$ leading to the definition of curvilinear derivatives and invariant time derivative

2 $${\nabla }_{i}{T}_{j}^{k}={\partial }_{i}{T}_{j}^{k}+{{\rm{\Gamma }}}_{im}^{k}{T}_{j}^{m}-{{\rm{\Gamma }}}_{ij}^{m}{T}_{m}^{k}$$

3 $$\dot{\nabla }{T}_{j}^{i}=\frac{\partial {T}_{j}^{i}}{\partial t}-{V}^{k}{\nabla }_{k}{T}_{j}^{i}+{\dot{{\rm{\Gamma }}}}_{k}^{i}{T}_{j}^{k}-{\dot{{\rm{\Gamma }}}}_{j}^{k}{T}_{k}^{i}$$

where the symbol ${\dot{{\rm{\Gamma }}}}_{a}^{b}$ is defined for the moving surfaces as ${\dot{{\rm{\Gamma }}}}_{a}^{b}={\nabla }_{a}{V}^{b}-C{B}_{a}^{b}$ and *B*_*ab*_ = ***N***∇_*a*_***S***_***b***_ is the curvature tensor. Definitions (1–3) along with the time derivatives of integrals form basics of calculus for moving surfaces and are necessary tools for derivation of dynamic equations^29^.

As far as we have published exact derivation few times already^28,29^, to avoid repetition but keep self-consistence, we provide generic equations for two-dimensional surface dynamics here and give only introduction to the derivation. The derivation follows from the minimal action principal of the Lagrangian:

4 $$L={\int }_{S}\,\frac{{\rho }_{S}{V}^{2}}{2}dS-{\int }_{{\rm{\Omega }}}\,ud{\rm{\Omega }}$$

where *u* is a potential energy density of a field defined on the volume Ω and *ρ*_*S*_ is the surface mass density. Details about the surface mass density is given below in the results section. If there are *n* ‘micro’ fields lived on the volume, then due to additivity of potential energies

$$u=\sum _{i=1}^{n}\,{u}_{i}$$

One may represent the potential energy density as a negative internal pressure applied on the surface, then ${u}_{i}=-\,{p}_{i},p=\sum \,{p}_{i}$ and the Lagrangian can be rewritten as:

5 $$L={\int }_{S}\,\frac{{\rho }_{S}{V}^{2}}{2}dS+{\int }_{{\rm{\Omega }}}\,pd{\rm{\Omega }}$$

Trivial boundary condition dictates that in the absence of the shape dynamics the surface mass must be conserved, therefore the first boundary condition leads to:

6 $$\dot{\nabla }{\rho }_{S}+{\nabla }_{i}({\rho }_{S}{V}^{i})={\rho }_{S}C{B}_{i}^{i}$$

(6) is continuity equation rewritten for curved surfaces, for derivations see refs^28,29^.

According to the time derivative of the space integral theorem, a variation of the potential energy term is $\delta {\int }_{{\rm{\Omega }}}\,pd{\rm{\Omega }}={\int }_{{\rm{\Omega }}}\,\partial p/\partial td{\rm{\Omega }}+{\int }_{S}\,pCdS$^28,29^. The normal term of the variation of the kinetic energy $\delta {\int }_{S}\,\rho {V}^{2}/2dS$ leads to ${\int }_{S}\,\rho C(\dot{\nabla }C+2{V}^{i}{\nabla }_{i}C+{V}^{a}{V}^{b}{B}_{ab})dS$ and therefore

7 $$\begin{array}{c}{\int }_{S}\,\rho C((\dot{\nabla }C+2{V}^{i}{\nabla }_{i}C+{V}^{a}{V}^{b}{B}_{ab})+p)dS=-\,{\int }_{{\rm{\Omega }}}\partial p/\partial td{\rm{\Omega }}\\ {\partial }_{\alpha }({V}^{\alpha }((\dot{\nabla }C+2{V}^{i}{\nabla }_{i}C+{V}^{a}{V}^{b}{B}_{ab})+p))=-\partial p/\partial t\end{array}$$

Here we used *C* = *N*_*α*_*V*^*α*^, where *N*_*α*_ is the *α* component of the surface normal, and applied the Gauss theorem to the left hand side of the integral equation, converting the surface integral to the space integral. The tangential term of the kinetic energy variation is ${\int }_{S}\,{\rho }_{S}{V}_{i}(\nabla {V}^{i}+{V}^{j}{\nabla }_{j}{V}^{i}-C{\nabla }^{i}C-C{V}^{j}{B}_{j}^{i})dS$ and it must be equal to the term coming from the tangential gradient of the surface pressure, which can be modeled as ${\int }_{S}-\,{V}_{i}{N}^{\alpha }{\nabla }^{i}{p}_{\alpha }dS$ (here *p*_*α*_ = *pN*_*α*_)^28,29^ and hence

8 $${\rho }_{S}(\nabla {V}^{i}+{V}^{j}{\nabla }_{j}{V}^{i}-C{\nabla }^{i}C-C{V}^{j}{B}_{j}^{i})=-\,{N}^{\alpha }{\nabla }^{i}{p}_{\alpha }$$

The combination of equations (6–8) gives the set for closed, two-dimensional surface dynamics. The equation (6) displays how the surface mass density may change when the surface moves and (7 and 8) show the motion in the normal and the tangent directions. The equations are complete set for the surface dynamics, as far as have four *ρ*_*S*_, *C*, *V*_1_, *V*_2_ unknowns and four differential equations. All information about how internal processes may effect on the surface dynamics is stored in the surface pressure term, which can be subject of modeling dependently on the nature of the problem. Because the Lagrangian (5) is invariant and the variation is taken by tensor calculus the equations are fully covariant.

## Results

Before we start formal derivations, we should give a formulation of the problem. We assume following scenario: a freely falling droplet in a gravity is bouncing vertically from a super-hydrophobic substrate, where the substrate itself is freely rising and falling surface. Find a shape dynamics of the droplet if the tangential velocities compared to the normal velocity, interaction between the droplet and the substrate, internal friction and path instabilities are all negligibly smalls. Note that according to experiments internal friction has insignificant effect on shape dynamics of droplets^2^. To tackle the problem one may numerically solve the Navier-Stokes equations^23–25^ that has already proven to be difficult (not to mention that the Navier-Stokes equations follow from simplifications of our surface dynamics equations)^28^. Instead, we use dynamic nonlinear equations for moving two-dimensional surfaces^28–30^ (referred as DVS equations throughout the text) and solve it analytically, therefore producing very short solution.

For the surface modeling we assume following characterization: the droplet is continuum medium of molecules and has a surface boundary. The surface boundary is continuum medium too, but is made from fast diffusing layer. Formally, molecules from the droplet also diffuse with an environment, though the diffusion of surface molecules is quicker because they are in direct chemical contact with the environment. Technically, it means that the surface is transient layer phase separated from the internal droplet and the environment. Given this, we assign *ρ*_*S*_ to the surface mass density, while the droplet mass density is designated by *ρ*. Because the surface is continuum medium the surface mass density directly implies the thickness of the ‘diffusive’ layer. For simplicity, we assume that a friction between the surface and the droplet is negligibly small, so that the surface does not slip on the droplet. In other words, we assume single surface formalism, though generalization for many surfaces is not conceptually difficult and can explain buckling of the surface when the droplet evaporates. Though buckling is entirely different phenomena and we do not address it in this paper. Note that invoking many surfaces formalism would also provoke concept of internal friction directly linked to a visco-elastic effect. Here, we ignore viscosity of the droplet because for water, according to experiments, viscosity has low or no effect on shape dynamics of water drops^2^. Therefore limitations by single surface should be sufficient, though it might not be enough for description of more viscous drops.

### Dynamic system: a surface

The formalism leading to the derivation of the differential variation of the surface (DVS) equations^28,29^, for freely falling droplets, generalizes Eulerian representation of fluid dynamics and in contrast to Navier-Stokes, as it is demonstrated in this paper, is analytically solvable. The formalism is fully covariant and its analytic as well as numerical solutions qualitatively exactly reproduces the surface motion.

As far as the body falls freely in the gravity, we need to add gravitation to the equations of the two-dimensional surface motion and therefore, the DVS equations^28–30^ should be rewritten accordingly. The Lagrangian of the motion reads:

9 $$L={\int }_{S}\,\frac{{\rho }_{S}{V}^{2}}{2}dS+{\int }_{{\rm{\Omega }}}\,(p-\rho gh)d{\rm{\Omega }}$$

where *ρ*_*S*_ is the surface mass density, ***V*** is the surface velocity, *p* is an internal pressure across the surface, *ρgh* is a hydrodynamic pressure applied by the gravity and *ρ* is the drop mass density. Note that the hydrodynamic pressure is added by negative sign because it is external one.

**Figure 1:**
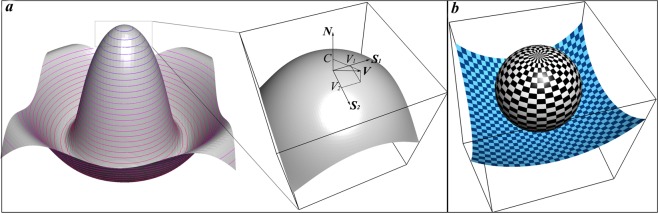
(**a**) Schematic drawing of the *z* = sin*R*/*R* surface and the illustration of the surface velocity with the normal and tangential components. The surface velocities *C*, *V*_1_, *V*_2_ for the point at the top are shown by arrows. The surface *z* = sin*R*/*R* is taken because it is reminiscent of the liquid substrate dynamics when the falling drop jumps. (**b**) Graphical illustration of a droplet sitting on a super hydrophobic substrate. Equilibrium shape of a droplet in a rest is a sphere.

The variation of the Lagrangian (9) modifies the DVS equations so that the gravitation is taken into account. As far as DVS equations are already derived^28,29^, instead of taking brute mathematical steps, we just mention how gravitational term *ρgh* modifies final equations that reads:

10 $$\begin{array}{c}\dot{\nabla }{\rho }_{S}+{\nabla }_{i}({\rho }_{S}{V}^{i})={\rho }_{S}C{B}_{i}^{i}\\ {\partial }_{\alpha }({V}^{\alpha }({\rho }_{S}(\dot{\nabla }C+2{V}^{i}{\nabla }_{i}C+{V}^{i}{V}^{j}{B}_{ij})+p-\rho gh))=-\,{V}^{\alpha }{\partial }_{\alpha }(p-\rho gh)\\ {\rho }_{S}(\dot{\nabla }{V}^{i}+{V}^{j}{\nabla }_{j}{V}^{i}-C{\nabla }^{i}C-C{V}^{j}{B}_{j}^{i})=-\,{N}^{\alpha }{\nabla }^{i}{p}_{\alpha }\end{array}$$

where *p*_*α*_ = (*p* − *ρgh*)*N*_*α*_, *α* = 1, 2, 3. As we have already mentioned above the first equation is a generalization of the conservation of mass, the second and the third equations display a motion in the normal and the tangent directions of the closed surface, and all satisfy conservation of mass and energy. $\dot{\nabla }=\partial /\partial t-{V}^{i}{\nabla }_{i},\,i=1,2$ is the curvilinear, invariant time derivative; ∇_*i*_ is the curvilinear derivative; *C* is the surface normal velocity; *V*_*i*_ is the component of the surface tangent velocity; *V*^*α*^ stands for ambient component for the surface velocity; *N*^*α*^ is the component of the unit surface normal; *B*_*ij*_ is the curvature tensor and ${B}_{i}^{i}$ is the mean curvature. The surface velocities are illustrated on Fig. 1. Repeated indexes indicate the Einstein summation convention. The equations (10) are covariant and are valid not only for capillary surfaces, but for moving surfaces of molecules too^28^.

Note that the equations (10) contradict Newton laws. According to Newton laws if there is no potential energy acting on the surface (no force), then the surface is in the rest or has constant surface velocities in the inertial reference frame. In contrast, what we find is that if one removes potential fields, i.e. sets *ρgh* and *p* naught in the equations, then one should expect that a solution to the equations must be constant normal *C* and constant tangent *V*_*i*_ velocities. It is easy to check that the constant surface velocity indeed satisfies the equations of motions (10) for freely floating droplets, but they are not the only solutions. Therefore, the DVS equations (10) predict that freely floating water drops will continue shape dynamics (if they were moving *a priori*) with retaining non-linearity even in the case when no potential field (no force) acts on them.

### Simplifications

(10) remains numerically unsolved. We give simplifications in this subsection and solve simplified equations numerically and analytically. We assume that the tangent components of the surface velocity is infinitesimally small compared to normal velocity and hence the tangential gradient of the surface pressure can be modeled as negligibly small:

11 $$-{N}^{\alpha }{\nabla }^{i}{p}_{\alpha }=0$$

This approximation is acceptable because in the most cases the surface pressure acts in the normal direction so that the pressure gradient in the tangent directions is zero. As we have already shown in our previous works^28,29^, the second equation significantly simplifies if the surface is homogeneous and can be described with time invariable surface tension *σ*, then

12 $${\rho }_{S}(\dot{\nabla }C+2{V}^{i}{\nabla }_{i}C+{V}^{i}{V}^{j}{B}_{ij})=\sigma {B}_{i}^{i}$$

The equation (12) is commonly known as the dynamic fluid film equation for the surface normal motion and has been reported before^20,31^. Using (12) in (10), we end up with

13 $${\partial }_{\alpha }[{V}^{\alpha }(\sigma {B}_{i}^{i}+p-\rho gh)]=-\,{V}^{\alpha }{\partial }_{\alpha }(p-\rho gh)$$

The equation (13) is a solution for the second equation indicating the surface motion in the normal direction. We now assume that deformations along tangent directions compared to normal ones are negligibly small, then equations (10) with condition (11) simplify as:

14 $$\frac{\partial {\rho }_{S}}{\partial t}={\rho }_{S}C{B}_{i}^{i}$$

15 $${\partial }_{\alpha }[{V}^{\alpha }({\boldsymbol{\sigma }}{B}_{i}^{i}+p-{\boldsymbol{\rho }}gh)]=-\,{V}^{\alpha }{\partial }_{\alpha }(p-{\boldsymbol{\rho }}gh)$$

16 $${{\boldsymbol{\rho }}}_{S}\frac{\partial C}{\partial t}={\boldsymbol{\sigma }}{B}_{i}^{i}$$

where (16) comes from (12) with assumption that tangent velocities are infinitesimally small. Note that, in this case, the (15) is the solution of (14, 16).

When the droplet touches the ground (the substrate surface), for very short interval of time, the gravity becomes compensated by a substrate surface reaction forces, so that, the drop instantaneously comes in equilibrium with the substrate and passes instantaneous equilibrium shape. The equilibrium shape satisfies conditions: *C* = 0, ∂_*α*_*V*^*α*^ = 0 and ∂(*p* − *ρgh*)/∂*t* = 0, then solutions to (14, 15) are:

17 $${\rho }_{S}={\rho }_{0}=const$$

18 $${B}_{i}^{i}=-\,\frac{p-\rho gh}{\sigma }$$

The equation (17) dictates that water molecules are homogeneously distributed on the surface, and (18) shows that the shape adopts constant mean curvature. Water droplets are closed and compact surfaces, therefore by the Alexandrov theorem^32^ the shape with constant mean curvature must be a sphere. Therefore, instantaneous equilibrium shape of the droplet as well as the droplet sitting on a super hydrophobic substrate is indeed perfect sphere^1^.

### Infinitesimal linear models

Before we proceed further, note that (14–17) are consistent with existing infinitesimal models^21,22^. Indeed, let *ρ*_0_ be equilibrium surface density and assume that both *C* and ${B}_{i}^{i}$ are infinitesimally small, so that linearized conservation of mass (14) reads ∂*ρ*_*S*_/∂*t* = 0 with the solution *ρ*_*S*_ = *ρ*_0_. This indicates that for small enough *C*, the density of the diffusive layer remains constant and infinitesimal models, in the limit of small oscillations and small mean curvature, are consistent with our framework.

Furthermore, if the mean curvature ${B}_{i}^{i}$ is the time dependent function, then according to calculus of moving surfaces

19 $$\frac{\partial {B}_{i}^{i}}{\partial t}={\rm{\Delta }}C+C{B}_{ij}{B}^{ij}$$

where Δ = ∇_*i*_∇^*i*^ is the surface Laplacian^31^. For surfaces with vanishing mean curvature, *B*_*ij*_*B*^*ij*^ becomes negative twice the Gaussian curvature *K* ^20^. Therefore, by differentiating (16) linearized acceleration becomes

$$\frac{{\partial }^{2}C}{\partial {t}^{2}}=\gamma ({\rm{\Delta }}C-2KC)$$

where *γ* = *σ*/*ρ*_0_. In the limit of planar cut off, i.e for flat equilibrium configurations, the Gaussian curvature is zero *K* = 0, and infinitesimal deformations are governed by the wave equation:

20 $$\frac{{\partial }^{2}C}{\partial {t}^{2}}=\gamma {\rm{\Delta }}C$$

With the sign convention, the equation (20) is exactly a case for linear models and is consistent with previous results^21,22^.

### Dynamic solutions

Now we proceed with extracting approximate analytic solutions for (14–16) equations and give numerical solution for (14, 16) equation set.

After the droplet spends some energy on the surface deformation, taking into account that it found instant equilibrium shape it jumps again, but since it has already found equilibrium, which is a sphere, it will start fluctuations around the equilibrium shape. Since, the shape oscillates near to equilibrium; we can suggest that according to (18) the term $\sigma {B}_{i}^{i}+p-\rho gh$ becomes infinitesimally small constant, therefore (15) can be modified as

21 $$\begin{array}{ccc}{\partial }_{\alpha }[{V}^{\alpha }(\sigma {B}_{i}^{i}+p-\rho gh)] & = & -\,{V}^{\alpha }{\partial }_{\alpha }(p-\rho gh)\\  & = & -({\partial }_{\alpha }[{V}^{\alpha }(p-\rho gh)]-(p-\rho gh){\partial }_{\alpha }{V}^{\alpha })\\ {\partial }_{\alpha }[{V}^{\alpha }(\sigma {B}_{i}^{i}+2(p-\rho gh))] & = & (p-\rho gh){\partial }_{\alpha }{V}^{\alpha }\end{array}$$

Since $\sigma {B}_{i}^{i}$ cannot be zero and its combination with the surface and hydrodynamic pressure is quasi infinitesimally small constant, then the solution to (21) is

22 $${\partial }_{\alpha }{V}^{\alpha }=0$$

**Figure 2:**
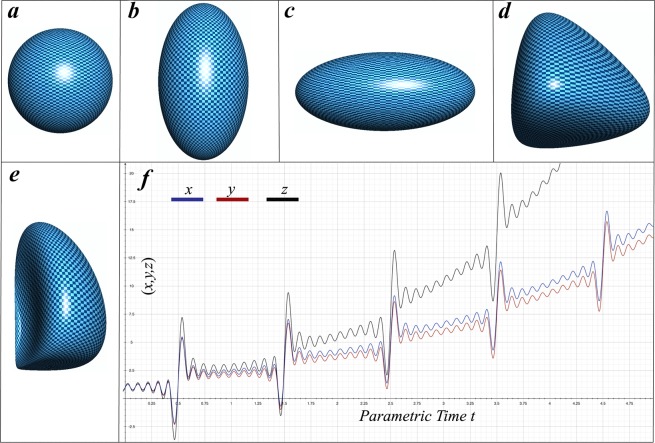
Illustration of analytically solved shapes. From left to right: (**a**) sphere with unit radius ***R***_**0**_ = 1 (initial condition of (24) solution), (**b**) prolate shape drop ***R*** = (sin*ϕ* sin*θ*, 0.86sin*ϕ* cos*θ*, 1.75cos*θ*) (solution (24) at some given time), (**c**) oblate shape ***R*** = (sin*ϕ* sin*θ*, 1.72sin*ϕ* cos*θ*, 0.75cos*θ*) (solution (24) at other moment), (**d)** non-prolate/oblate shape ***R*** = (*e*^sin*ϕ* sin*θ*^, *e*^sin*ϕ*cos*θ*^, *e*^cos*θ*^) (solution (25) at some given time), (**e**) summation of (**a**–**d**) shapes indicate development of continues singularities (left hand side of the droplet). Panel (f) displays plot of analytic solution and indicates how spacial coordinates (*x*, *y*, *z*) non-periodically, asymmetrically and with growing amplitudes fluctuate in time. The graph also indicates that (*z*) direction (black graph) diverges from (*x*, *y*) rapidly while (*x*) (blue graph) separates from (*y*) slowly, meaning: the drop will more rapidly develop singularities in (*z*) direction while will keep stability in (*x*, *y*) directions for relatively long time.

This is the continuity condition in the Navier-Stokes equations for incompressible fluids^33^. However, setting $\sigma {B}_{i}^{i}+p-\rho gh$ as infinitesimally small constant is an approximation and therefore, (22) is not an exact solution. (22) shows that zero divergence of the surface velocity is the near equilibrium solution of the DVS for water drops under the gravity. (22) can be trivially handled by the ***V*** = *k****R***/*R*^3^ function (where *k* is some constant and ***R*** is the position vector), which corresponds to the sphere in equilibrium stationary case. Therefore, in the (0 ≤ *θ* ≤ 2*π*, 0 ≤ *ϕ* ≤ *π*) spherical coordinates, assuming designation ***S*** = (sin*ϕ* sin*θ*, sin*ϕ* cos*θ*, cos*θ*) the solution reads:

23 $$\frac{\partial {\boldsymbol{R}}}{\partial t}={\omega }_{\xi }{R}_{\xi }{{\boldsymbol{S}}}^{{\boldsymbol{\xi }}}$$

where *ω*_*ξ*_, *ξ* = *x*, *y*, *z* are some frequency like functions and for the sake of simplicity we consider them as constants. ***S***^***ξ***^ is the base vector of ***S*** and *R*_*ξ*_ is the projection of the position vector in the *ξ* direction (for example ***S***^***x***^ = (sin*ϕ* sin*θ*, 0, 0), ***S***^***y***^ = (0, sin*ϕ* cos*θ*, 0), ***S***^***z***^ = (0, 0, cos*θ*) and *R*_*ξ*_***S***^***ξ***^ = *R*_*x*_***S***^***x***^ + *R*_*y*_***S***^***y***^ + *R*_*z*_***S***^***z***^). Trivially, (22, 23) leads to the solutions: for some *R*_*ξ*_ constants with initial condition ***R***_***t***_ = _**0**_ = ***R***_**0**_ = *R*_0_***S*** and *R*_*ξ*_ = *R*_*ξ*_ (*t*) time variable function, reading respectively:

24 $${\boldsymbol{R}}={{\boldsymbol{R}}}_{{\bf{0}}}+{\omega }_{\xi }{R}_{\xi }t{{\boldsymbol{S}}}^{{\boldsymbol{\xi }}}$$

25 $${\boldsymbol{R}}={R}_{0\xi }{e}^{{\omega }_{\xi }t{{\boldsymbol{S}}}^{{\boldsymbol{\xi }}}}$$

where ${e}^{{\omega }_{\xi }t{{\boldsymbol{S}}}^{{\boldsymbol{\xi }}}}$ is defined as the vector components of which are exponents of frequency times time and the spherical coordinate: ${e}^{{\omega }_{\xi }t{{\boldsymbol{S}}}^{{\boldsymbol{\xi }}}}=({e}^{{\omega }_{x}t\sin \varphi \sin \theta },{e}^{{\omega }_{y}t\sin \varphi \cos \theta },{e}^{{\omega }_{z}t\cos \theta })$. (Figure 2a–e) are a graphical representation of (24, 25) solutions. In addition to (24, 25), according to the vector calculus the analytic solution to (22) is

26 $${\boldsymbol{V}}=-\,{\boldsymbol{\nabla }}\times  {\mathcal L} $$

where ${\mathcal L}$ vector has the same dimension as angular velocity. Suggesting that ${\mathcal L}$ should behave same way as ***R***, with conserving full generality, the solution (26) can be rewritten as

27 $$\begin{array}{rcl}\frac{\partial {\boldsymbol{R}}}{\partial t} & = & -{\boldsymbol{\nabla }}\times  {\mathcal L} \\ \frac{1}{{v}_{0}^{2}}\frac{\partial  {\mathcal L} }{\partial t} & = & {\boldsymbol{\nabla }}\times {\boldsymbol{R}}\end{array}$$

where *v*_0_ is some constant wave propagation velocity. After taking the curl of (27) and using the curl of the curl (**∇** × **∇**) identity one ends up with a wave equation for the position vector:

28 $$\frac{1}{{v}_{0}^{2}}\frac{{\partial }^{2}{\boldsymbol{R}}}{\partial {t}^{2}}-{\nabla }^{2}{\boldsymbol{R}}=0$$

According to curl of curl identity ∇ × (∇ × ⋅) = ∇(∇⋅)−∇^2^⋅, applying this to (27) one obtains $\nabla (\nabla {\boldsymbol{R}})-{\nabla }^{2}{\boldsymbol{R}}=$ $-\,1/{v}_{0}^{2}{\partial }^{2}{\boldsymbol{R}}/\partial {t}^{2}$. Taking into account (22) ∇***R*** = *const*, therefore first term vanishes and one ends up with (28).

Taking into account that as the time evolves all spatial directions become linearly independent *R*_*ξ*_ = *ψ*_*ξ*_ (*ξ*, *t*) (where *ξ* = *x*, *y*, *z* and *ψ* is some function), then (28) transforms as one dimensional wave equation $1/{v}_{0}^{2}{\partial }^{2}{\psi }_{\xi }/\partial {t}^{2}=$ ${\partial }^{2}{\psi }_{\xi }/\partial {\xi }^{2}$. Applying boundary *ψ*(0, *t*) = 0, *ψ*(*L*, 0) = 0 and initial conditions *ψ*(*ξ*, 0) = *f*(*ξ*), ∂*ψ*/∂*t*(*ξ*, 0) = *g*(*ξ*) one obtains a solution

$${{\boldsymbol{\psi }}}_{\xi }(\xi ,t)=\sum _{m=1}^{\infty }\,\frac{2}{L}({\int }_{0}^{L}\,{\boldsymbol{\psi }}(\xi ,0)\,\sin \,\frac{m\pi \xi }{L}d\xi )\cos (\frac{{v}_{0}m\pi t}{L})\sin (\frac{{v}_{0}m\pi t}{L})$$

Taking into account that at the initial condition ***R***(*t* = 0) = ***R***_**0**_ the water drop is a sphere with the radius *R*_0_, then the solution can be written as

29 $${\boldsymbol{R}}={{\boldsymbol{R}}}_{0}+{\psi }_{\xi }{{\boldsymbol{S}}}^{{\boldsymbol{\xi }}}$$

The solution (29) precisely explains oblate-prolate oscillation of the drop observed in experiments. (Figure 2f) shows dependence of the position vector components (*x*, *y*, *z*) on the parametric time and is plotted based on the linear combination of the (24, 25, 29) solutions. The graph is plotted for *L* = 1, *f*(*ξ*) = *ξ* parameters on the *ϕ* = *θ* = *π*/4 spherical coordinate and is designated to show phenomenological dependence of the point spacial coordinates (*x*, *y*, *z*) on the parametric time. The solution clearly indicates non-periodic and asymmetric behavior in all directions. *z* direction diverges from *x*, *y* rapidly while *x*, *y* diverges slowly from each other. Consequently, the drop more rapidly develops singularities in one direction while keeps stability in others for relatively long time. After long enough time, there is no overlap in amplitudes resulting in drop division.

**Figure 3:**
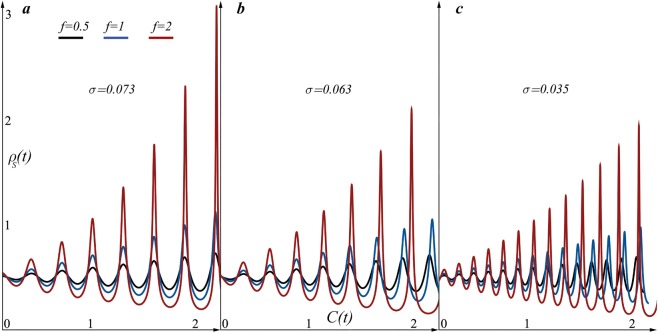
The numerical solution in (*ρ*_*S*_, *C*) phase space of (13, 15) equations reminiscent to the analytic solutions of the (14) equation. Black, blue and red lines are plotted for different amplitude values of *C* = *f*sin(*πt*): *f* = 0.5 black, *f* = 1 blue and *f* = 2 red. The initial condition for the interface velocity *C* is set to be zero *C*(0) = 0 and the initial value for the surface mass density for initially flat configurations is fixed *ρ*_*S*_ = 0.5. The mean curvature ${B}_{i}^{i}$ is set to be one corresponding to the sphere with unit radius. (a–c) Panels display simulations for three different tensions.

We have also solved (14, 16) equations numerically by the 4th generation Runge-Kutta method for 0.0001 time lapse and interval of 30 nominal parametric time. The numerical solution in (*ρ*_*S*_, *C*) phase space (Fig. 3) displays nonlinear thickening with growing amplitudes of diffusive layer, once again indicates nonlinear effects of nonperiodic/asymmetric motion and the growing amplitude in the surface density oscillations, and qualitatively well agrees to experimental data^1–19^.

In addition, to indicate how different values of surface tensions may effect on the surface density oscillations, we have plotted (*ρ*_*S*_, *C*) phase space for three different surface tensions (see Fig. 3). The initial value of the surface tension (Fig. 3a) *σ* = 0.073*N*/*m* is set to be the standard value for water drops. (Fig. 3b) is plotted for the surface tension of the inkjetprinted picoliter droplet *σ* = 0.063*N*/*m* and (Fig. 3c) displays oscillations for the microliter droplet with tension *σ* = 0.035*N*/*m*. The different values for the surface tensions is taken from the ref.^34^. (Fig. 3) shows that decrease in the surface tension decreases oscillation amplitudes and increases frequency.

Since (24, 25, 29) are particular solutions, then their linear combination is a general solution. Therefore, it predicts non-linear oscillations among spherical, oblate/prolate and non-oblate/prolate shapes, tendency towards increasing the radius, and inducing shape instabilities by forming singularities, and with combination of the numerical solution to (14, 16) indicates the growing amplitude in the oscillations of the surface mass density, meaning increasing the mass instabilities in the diffusive layer of the surface (Figs 2f and 3). Mass instabilities in the diffusive layer and linear combination of the (24, 25, 29) solutions ultimately lead to a development of singularities in the drop, which may induce drop division.

### Relevance to experiments

To explain shape dynamics of axisymmetrically deformed drop Rayleigh^35^ proposed expansion of absolute value of radius vector *R* in Legendre polynomials *L*_*n*_ so that $R(\theta ,t)=\sum {a}_{n}(t){L}_{n}(t)$ where *θ* is the polar angle, *a*_*n*_ is the time dependent amplitude coefficients and *n* is so called the mode number. Later on Lamb^36^ suggested that for a freely oscillating drop with small amplitude of oscillation the coefficients can be estimated as

$${a}_{n}(t) \sim {e}^{-{\beta }_{n}t}\,\sin ({\omega }_{n}t+{\varphi }_{n})+{b}_{n}$$

where *ω*_*n*_ = 2*π*/*T* is an oscillation eigenfrequency with decaying period *T*, *ϕ*_*n*_ is the phase angle, *b*_*n*_ is the eccentricity of the drop, which may arise from oblation of the droplets due to hydrodynamic effects, *β*_*n*_ is the dumping rate of the *n* mode. We refer to this model as Rayleigh’s exponentially decaying model. The model was successfully used to describe recent experiments^34,37–40^.

The eigenfrequency *ω*_*n*_ depends on the liquid density *ρ* and the surface tension *σ*, therefore *a*_*n*_ = *a*_*n*_(*ρ*_*S*_) and hence in the first approximation ${a}_{n} \sim {\rho }_{S}$. On other hand, in the first approximation *a*_*n*_ is proportional to the surface interface velocity *C*, therefore (*ρ*_*S*_, *C*) phase space has to mimic the Rayleigh’s exponentially decaying model.

**Figure 4:**
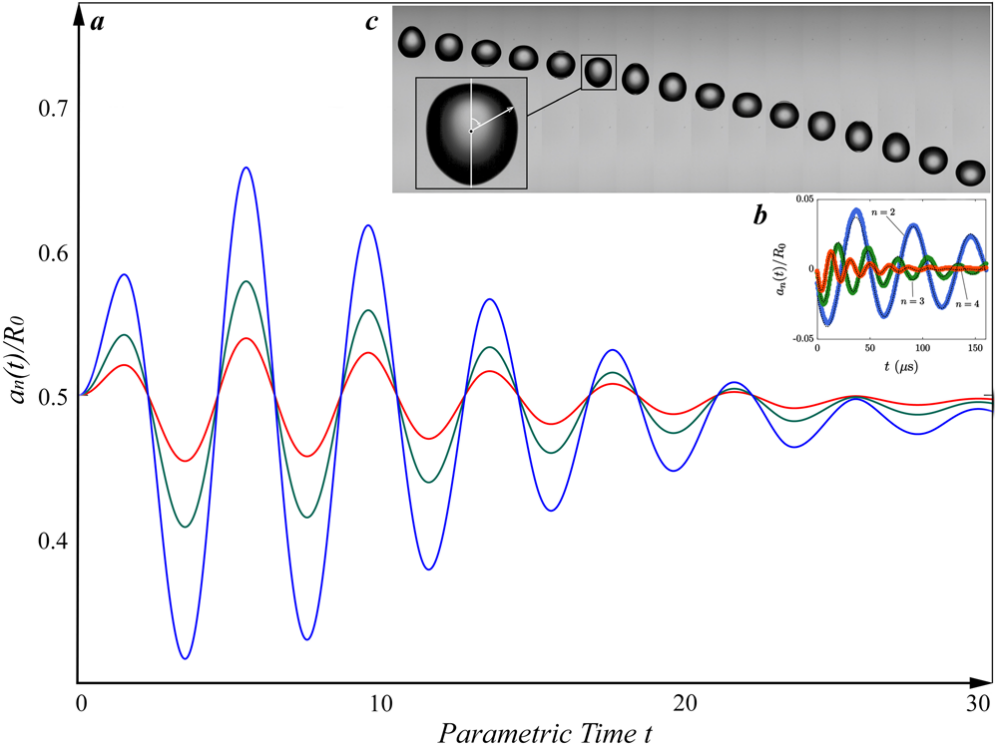
(**a**) The numerical simulation of the dependence of amplitudes on time. (**a**) Red, green and blue curves correspond to different *f*_*n*_(0) interface velocity amplitudes and produce remarkable morphological similarities with (**b**) experimentally observed decaying oscillation amplitudes of water drops. The panel (c) provides photographs of micro sized droplets shape oscillations at different microseconds. Experimentally observed shapes well agree to our analytic and numerically solved shapes. (**b**,**c**) Experimental data is obtained from the ref.^34^.

Indeed, for comparison to previous experimental works we mention that in our numerical calculations for interface velocity amplitudes we used *f* = *a*sin(*πt*). However, instead of harmonic *f* if one uses Rayleigh’s decaying amplitude model, which can be expressed as *f*_*n*_(*t*) = *f*_*n*_(0)exp(−*t*/*T*) sin(2*πt*/*T* + *ϕ*) + *b*_*n*_^35,36^, then one should expect that (*ρ*_*S*_, *t*) would mimic overall morphology of experimentally obtained decaying amplitude oscillation of droplets mean radius. Indeed, as the (Fig. 4) shows plotted graphs for different *f*_*n*_: *f*_*n*_(*t*) = 0.5exp(−*t*/5) sin(*πt* + *π*/4) red, *f*_*n*_(*t*) = exp(−*t*/5) sin(*πt*/2 + *π*/4) green and *f*_*n*_(*t*) = 2exp(−*t*/2) sin(*πt*/2 + *π*/4) blue curves indicate similarity of overall morphology of numerically calculated (Fig. 4a) and experimentally observed (Fig. 4b)^34^ graphs. Note that in the (Fig. 4a) the surface mass density is modeled as *ρ*_*s*_ = *ka*_*n*_(*t*)/*R*_0_, where *k* is a coupling constant and is set as unit in the numerical solution, *a*_*n*_ is the oscillation amplitude of mode *n* and *R*_0_ is the initial radius of the spherical droplet. The numerical solution for the (*a*_*n*_(*t*)/*R*_0_, *t*) space were obtained by ode45 numerical solver. These calculations do imply that if one carefully picks and fits parameters in theoretical (24, 25, 29) and numerical calculations of (14, 16), then one will reproduce experimentally observed dependence of oscillation amplitude on the time. For clarifications that are more rigorous it is necessary to be made theoretical calculations, experimental observations and parameter fitting simultaneously, which is not possible now but is left out as a source for future collaborations for experimental suggestions and clarifications.

## Discussion

Despite neglecting uniform translational motion of the drop (one may argue that for sufficiently small periods of time the inertial effects on drop shape motion is negligibly small), we might still speculate why oscillating surfaces can lunch water drop at higher speed, than hard sphere^2^. Due to the generality of the DVS equations, an oscillating surface undergoes the same shape motion at the interface of substrate/droplet as water drops. If frequencies of the substrate surface and water drop matched, within an order of magnitude, then a resonance effect takes place and the drop will be launched by higher speed. We should also note that according to (12–18) and (21) equations gravitational term *ρgh* has no effect on governing shape equations. Gravity has no role therefore surface motion would be the same if one would remove gravity. This explains why water drops can move against gravity^7^ on oscillating super hydrophobic substrate.

To conclude we have proposed a system of nonlinear equations to describe the nonlinear features of the dynamics of two-dimensional surfaces, with large deformations and large variations in density, both spatial and temporal^28–30^. Analytic solutions to simplified version of two-dimensional surface equations in gravity describe freely falling water drop’s shape dynamics and precisely explain why the shape non-linearly oscillates among prolate/oblate and non-prolate/oblate forms and display a wide range of shape instabilities. Surprisingly, despite the fact that the proposed system purposefully disregards the tangential components of surface velocities, the solution qualitatively explains experimentally observed shape dynamics^1–9^. By this analytic solution, we have reproduced numerically solved Navier-Stokes equations results^24^, but avoided intensive modeling for the surface pressure, which would be necessary for numerically solving Navier-Stokes. We have shown that the continuity equation is an approximate solution of the DVS equations, which, in contrast to Navier-Stokes, can be trivially handled in this particular case. In addition, we have shown that linear models leading to wave equations^21,22^ are generally correct for near to equilibrium approximations.

The numerical solutions of (14, 16) combined with analytical ones (24, 25, 29) directly imply that: the surface mass density is non-linearly increasing while the shape adopts diverse forms. Impact of this statement in biology is that: if one starts formation of cell from a mixture of organic molecules in a droplet and lets evolution of the shape dynamics by applying some potential field (like hydrophobic-hydrophilic interactions for instance), then the droplet will ultimately develop some membrane like structure (because surface mass density is nonlinearly increasing) and will adopt rich divers shapes. Such active dynamics may explain why cells got membranes^41^ and is in contradiction with generally thought idea that membranes were formed first.

## Data Availability

All derivations and numerical calculations are included in the text.

## References

[1] de Gennes, P.-G., Brochard-Wyart, F. & Quéré, D. *Capillarity and gravity*, 33–67, 10.1007/978-0-387-21656-0_2 (Springer New York, New York, NY, 2004).

[2] Raufaste C (2017). Superpropulsion of droplets and soft elastic solids. Phys. Rev. Lett..

[3] Chaudhury, M. K. & Whitesides, G. M. How to make water run uphill. *Science* **256**, 1539–1541 http://science.sciencemag.org/content/256/5063/1539 (1992). 10.1126/science.256.5063.1539 17836321

[4] Sumino Y, Magome N, Hamada T, Yoshikawa K (2005). Self-running droplet: emergence of regular motion from nonequilibrium noise. Phys. Rev. Lett..

[5] Daniel S, Chaudhury MK, de Gennes P-G (2005). Vibration-actuated drop motion on surfaces for batch microfluidic processes. Langmuir.

[6] Noblin X, Kofman R, Celestini F (2009). Ratchetlike motion of a shaken drop. Phys. Rev. Lett..

[7] Brunet P, Eggers J, Deegan RD (2007). Vibration-induced climbing of drops. Phys. Rev. Lett..

[8] Vollmer D, Butt H-J (2014). Shaping drops. Nature Physics.

[9] Richard D, Clanet C, Quéré D (2002). Contact time of a bouncing drop. Nature.

[10] Kellay, H. & Goldburg, W. I. Two-dimensional turbulence: a review of some recent experiments. *Reports on Progress in Physics* **65**, 845, http://stacks.iop.org/0034-4885/65/i=5/a=204 (2002).

[11] Greffier O, Amarouchene Y, Kellay H (2002). Thickness fluctuations in turbulent soap films. Phys. Rev. Lett..

[12] Rivera M, Vorobieff P, Ecke RE (1998). Turbulence in flowing soap films: Velocity, vorticity, and thickness fields. Phys. Rev. Lett..

[13] Van Nierop EA, Scheid B, Stone HA (2008). On the thickness of soap films: an alternative to frankel’s law. Journal of Fluid Mechanics.

[14] Tran T, Chakraborty P, Gioia G, Steers S, Goldburg W (2009). Marangoni shocks in unobstructed soap-film flows. Phys. Rev. Lett..

[15] Moulton DE, Pelesko JA (2010). Reverse draining of a magnetic soap film. Phys. Rev. E.

[16] Drenckhan W, Dollet B, Hutzler S, Elias F (2008). Soap films under large-amplitude oscillations. Philosophical Magazine Letters.

[17] Debrégeas G, Martin P, Brochard-Wyart F (1995). Viscous bursting of suspended films. Phys. Rev. Lett..

[18] Boudaoud A, Couder Y, Ben Amar M (1999). Self-adaptation in vibrating soap films. Phys. Rev. Lett..

[19] Gilet T, Bush JWM (2009). Chaotic bouncing of a droplet on a soap film. Phys. Rev. Lett..

[20] Grinfeld P (2010). Variable thickness model for fluid films under large displacement. Physical Review Letters.

[21] Isenberg, C. *The science of soap films and soap bubbles* (Dover Publications, New York, 1992).

[22] Chomaz, J. & Costa, M. Thin film dynamics. In *Free surface flows*, 45–99 (Springer, 1998).

[23] Tripathi MK, Sahu KC, Govindarajan R (2015). Dynamics of an initially spherical bubble rising in quiescent liquid. Nature Communications.

[24] Agrawal M, Premlata AR, Tripathi MK, Karri B, Sahu KC (2017). Nonspherical liquid droplet falling in air. Phys. Rev. E.

[25] Tripathi, M. K., Sahu, K. C. & Govindarajan, R. Why a falling drop does not in general behave like a rising bubble. *Scientific Reports* **4** (2014). 10.1038/srep04771 PMC3998034 24759766

[26] Eggers J, Dupont TF (1994). Drop formation in a one-dimensional approximation of the navier–stokes equation. Journal of Fluid Mechanics.

[27] Shi XD, Brenner MP, Nagel SR (1994). A cascade of structure in a drop falling from a faucet. Science.

[28] Svintradze, D. V. Moving manifolds in electromagnetic fields. *Frontiers in Physics* **5**, 37, http://journal.frontiersin.org/article/10.3389/fphy.2017.00037 (2017).

[29] Svintradze, D. V. Closed, two dimensional surface dynamics. *Frontiers in Physics* **6**, 136, https://www.frontiersin.org/article/10.3389/fphy.2018.00136 (2018).

[30] Svintradze, D. V. Micelles hydrodynamics. *arXiv preprint arXiv:1608.01491* (2016).

[31] Grinfeld, P. *Introduction to tensor analysis and the calculus of moving surfaces* (Springer, 2013).

[32] Alexandrov AD (1958). Uniqueness theorem for surfaces in the large. Leningrad Univ. 13, 19 (1958), 5–8, Amer. Math. Soc. Trans. (Series 2).

[33] Landau, L. & Lifshitz, E. In *Fluid mechanics*, second edition edn., https://www.sciencedirect.com/science/article/pii/B9780080339337500052 (Pergamon, 1987).

[34] Staat HJJ (2016). Ultrafast imaging method to measure surface tension and viscosity of inkjet-printed droplets in flight. Experiments in Fluids.

[35] Strutt, J. W. Vi. on the capillary phenomena of jets. *Proceedings of the Royal Society of London* **29**, 71–97 (1879).

[36] Lamb H (1881). On the oscillations of a viscous spheroid. Proceedings of the London Mathematical Society.

[37] Becker E, Hiller WJ, Kowalewski TA (1991). Experimental and theoretical investigation of large-amplitude oscillations of liquid droplets. Journal of Fluid Mechanics.

[38] Yang L (2014). Determination of dynamic surface tension and viscosity of non-newtonian fluids from drop oscillations. Physics of Fluids.

[39] Kremer J, Kilzer A, Petermann M (2018). Simultaneous measurement of surface tension and viscosity using freely decaying oscillations of acoustically levitated droplets. Review of Scientific Instruments.

[40] Hoath, S. D. *et al*. Oscillations of aqueous pedot:pss fluid droplets and the properties of complex fluids in drop-on-demand inkjet printing. *Journal of Non-Newtonian Fluid Mechanics* **223**, 28–36, http://www.sciencedirect.com/science/article/pii/S0377025715000993 (2015).

[41] Zwicker D, Seyboldt R, Weber CA, Hyman AA, Jülicher F (2016). Growth and division of active droplets provides a model for protocells. Nature Physics.

